# Evidence for two morphologically cryptic species of *Hysterolecitha* Linton, 1910 (Trematoda: Lecithasteridae) infecting overlapping host ranges in Moreton Bay, Australia

**DOI:** 10.1007/s11230-023-10092-6

**Published:** 2023-05-03

**Authors:** Berilin Duong, Thomas H. Cribb, Scott C. Cutmore

**Affiliations:** 1 School of Biological Sciences, The University of Queensland, St Lucia, QLD 4072 Australia; 2 Queensland Museum, Biodiversity and Geosciences Program, South Brisbane, QLD 4101 Australia

## Abstract

Integration of morphological and molecular approaches to species delineation has become an essential part of digenean trematode taxonomy, particularly when delimiting cryptic species. Here, we use an integrated approach to distinguish and describe two morphologically cryptic species of *Hysterolecitha* Linton, 1910 (Trematoda: Lecithasteridae) from fishes of Moreton Bay, Queensland, Australia. Morphological analyses of *Hysterolecitha* specimens from six fish species demonstrated a complete overlap in morphometric data with no reliable differences in their gross morphological characters that suggested the presence of more than one species. Distinctions in ITS2 rDNA and *cox*1 mtDNA sequence data for corresponding specimens suggested the presence of two forms. A principal component analysis on an imputed dataset showed clear separation between the two forms. These two forms are partially separated on the basis of their host’s identity. Therefore, we describe two morphologically cryptic species: *Hysterolecitha melae* **n. sp.** from three species of *Abudefduf* Forsskål and one species of *Parma* Günther (Pomacentridae), with the Bengal sergeant, *Abudefduf bengalensis* (Bloch), as the type-host; and *Hysterolecitha phisoni* **n. sp.** from species of Pomacentridae (including *A. bengalensis*), Pomatomidae and Siganidae, with the black rabbitfish, *Siganus fuscescens* (Houttuyn), as the type-host.

## Introduction

The Hysterolecithinae Yamaguti, 1958 (Lecithasteridae) is the second largest of the six lecithasterid subfamilies, comprising four genera, *Hysterolecitha* Linton, 1910, *Hysterolecithoides* Yamaguti, 1934, *Machidatrema* León-Règagnon, 1998, and *Thulinia* Gibson & Bray, 1979. Members of the Hysterolecithinae differ from those of other lecithasterid subfamilies in the possession of Juel’s organ and a uterine seminal receptacle (Gibson, 2002). The type-genus, *Hysterolecitha*, is the richest of the four hysterolecithine genera, comprising 22 recognised marine and freshwater species. Species of this genus are distinguished from those of *Hysterolecithoides*, *Machidatrema* and *Thulinia* by an anterior fusion of the excretory ducts, absence of filamented eggs, and a weakly developed sinus-sac.

Species of *Hysterolecitha* have been reported from fishes from 21 families and most have been reported with oioxenous host-specificity (Table 1). Notably, while some species have been reported to be stenoxenic or euryxenic (infecting several host species of a single family or multiple families, respectively) or have extensive geographical distributions, these broad specificities and distributions have yet be confirmed with molecular data. Just two species of *Hysterolecitha* have been reported from Australian waters, *H. heronensis* Bray, Cribb & Barker, 1993 and *H. nahaensis* Yamaguti, 1942. *Hysterolecitha heronensis* was described from the Philippine damsel, *Pomacentrus philippinus* Evermann & Seale (Pomacentridae), off Heron Island in the southern Great Barrier Reef (GBR), Australia, and was reported from four other species of *Pomacentrus* Lacépède on the GBR (Bray et al., 1993; Barker et al., 1994; Sun et al., 2012). *Hysterolecitha nahaensis* was originally described from the humbug damselfish, *Dascyllus aruanus* (Linnaeus), off Okinawa, Japan (Yamaguti, 1942), and has since been reported mainly from pomacentrid fishes from the GBR (Bray et al., 1993; Barker et al., 1994; Sun et al., 2012) and the South China Sea (King, 1964; Zhokhov et al., 2018), and rarely from other fish families in localities such as the Celebes Sea (Yamaguti, 1953) and the Mozambique Channel (Parukhin, 1989).

**Table 1:** Host information, host-specificity, and geographic distribution for the 22 recognised species of *Hysterolecitha* Linton, 1910.

**Species**	**Localities**	**Host species**	**Host-specificity**	**References**
*Hysterolecitha acanthuri* Annereaux, 1947	Off Mercedes, Philippines	Acanthuridae: *Acanthurus triostegus* (Linnaeus)	Oioxenous	Annereaux (1947)
*Hysterolecitha arii* Wang, 1982	Off Putian, China	Bagridae: *Tachysurus sinensis* Lacépède	Oioxenous	Wang (1982)
*Hysterolecitha blepsiae* Layman, 1930	Peter the Great Bay, Russia	Hemitripteridae: *Blepsias cirrhosus* (Pallas)	Oioxenous	Layman (1930)
*Hysterolecitha brasiliensis* de Oliveira, Amato & Knoff, 1988	Off Rio de Janeiro, Brazil	Mugilidae: *Mugil liza* Valenciennes	Oioxenous	de Oliveira et al. (1988); Knoff et al. (1997)
*Hysterolecitha chirocentri* Ku & Shen, 1964	Gulf of Tonkin, China	Chirocentridae: *Chirocentrus dorab* (Forsskål)	Oioxenous	Shen (1990)
*Hysterolecitha crassivesiculata* Bravo-Hollis, 1956	Off Puerto Vallarta, Mexico	Cirrhitidae: *Cirrhitus rivulatus* Valenciennes	Oioxenous	Bravo-Hollis (1956)
*Hysterolecitha elongata* Manter, 1931	Gulf of Mexico; Off Beaufort, North Carolina, United States; Off Espírito Santo, Brazil	Mugilidae: *Mugil cephalus* Linnaeus, *Mugil liza*	Stenoxenous	Manter (1931); Pearse (1949); Travassos et al. (1967); Overstreet (1973); Gomes et al. (1974)
*Hysterolecitha flaticaudata* Bilqees, Feroze & Shaukat, 2004	Off Karachi, Pakistan	Engraulidae: *Thryssa purava* (Hamilton)	Oioxenous	Bilqees et al. (2004)
*Hysterolecitha heronensis* Bray, Cribb & Barker, 1993	Off Heron Island and Lizard Island, Great Barrier Reef	Pomacentridae: *Pomacentrus amboinensis* Bleeker, *Pomacentrus moluccensis* Bleeker, *Pomacentrus nigromarginatus* Allen, *Pomacentrus philippinus* Evermann & Seale	Stenoxenous	Bray et al. (1993); Barker et al. (1994); Sun et al. (2012)
*Hysterolecitha indica* Mehra, 1969	Prayagraj, India (freshwater)	Channidae: *Channa punctata* (Bloch)	Oioxenous	Mehra (1969)
*Hysterolecitha indonesiana* Machida, 1996	Off Ambon Island, Indonesia	Mugilidae: *Mugil cephalus*	Oioxenous	Machida (1996)
*Hysterolecitha lintoni* Srivastava, 1939	Off Karachi, India	Ariidae: *Plicofollis dussumieri* (Valenciennes)	Oioxenous	Srivastava (1939); Chauhan (1953)
*Hysterolecitha nahaensis* Yamaguti, 1942	Off Heron Island, Great Barrier Reef; Off Okinawa and Tsushima Island, Japan; Off Nha Trang, Vietnam; Masirah Bay, Arabian Sea; Off Macassar, Indonesia; Mozambique Channel	Acanthuridae: *Acanthurus nigricans* (Linnaeus); Lobotidae: *Lobotes* sp.; Macrouridae: *Coryphaenoides striaturus* Barnard; Pomacentridae: 25 species; Scaridae: *Callyodon* sp.	Euryxenous	Yamaguti (1942); Yamaguti (1953); King (1964); Ichihara (1974); Parukhin (1976; 1989); Bray et al. (1993); Barker et al. (1994); Zhokhov et al. (2018)
*Hysterolecitha ophiocephali* Mehra, Kharoo & Dhar, 1985	Prayagraj, India (freshwater)	Channidae: *Channa punctata*	Oioxenous	Mehra et al. (1985)
*Hysterolecitha palani* Yamaguti, 1970	Hawaii, United States	Acanthuridae: *Acanthurus dussumieri* Valenciennes	Oioxenous	Yamaguti (1970)
*Hysterolecitha progonimus* Ku & Shen, 1964	Gulf of Tonkin, China	Abulidae: *Albula vulpes* (Linnaeus)	Oioxenous	Ku & Shen (1964); Shen (1990)
*Hysterolecitha rosea* Linton, 1910	Dry Tortugas, United States; Off La Chorrera, Panama; Bimini, The Bahamas; Off Mona Island and Guayanilla Bay, Puerto Rico; Off Jamaica; Off Belize; Off Pingtan Island, China	Acanthuridae: *Paracanthurus hepatus* (Linnaeus), *Acanthurus bahianus* Castelnau, *Acanthurus coeruleus* Bloch & Schneider; Mugilidae: *Mugil curema* Valenciennes; Sciaenidae: *Nibea albiflora* (Richardson)	Euryxenous	Linton (1910); Manter (1947); Vigueras (1958); Sogandares-Bernal (1959); Siddiqi & Cable (1960); Nahhas & Cable (1964); Fischthal (1977); Wang (1982); Dyer et al. (1985); Dyer et al. (1992)
*Hysterolecitha sogandaresi* Nahhas & Cable, 1964	Off Jamaica	Acanthuridae: *Acanthurus coeruleus*	Oioxenous	Nahhas & Cable (1964)
*Hysterolecitha soniae* León-Règagnon, Pérez-Ponce de León & Lamothe-Argumedo, 1997	Chamela Bay, Mexico	Kyphosidae: *Kyphosus ocyurus* (Jordan & Gilbert)	Oioxenous	León-Règagnon et al. (1997)
*Hysterolecitha teuthis* Nagaty, 1956	Off Hurghada (Ghardaga), Egypt	Siganidae: *Siganus spinus* (Linnaeus)	Oioxenous	Nagaty (1956)
*Hysterolecitha trilocalis* King & Noble, 1961	Off Goleta, California, United States	Gobiidae: *Gillichthys mirabilis* Cooper	Oioxenous	King & Noble (1961)
*Hysterolecitha vitellograndis* (Layman, 1930) Skrjabin & Guschanskaja, 1954	Peter the Great Bay, Russia; Toyama Bay and Sagami Bay, Japan; Off Fuzhou, China	Stromateidae: *Thamnaconus modestus* (Günther); Paralichthyidae: *Paralichthys olivaceus* (Temminck & Schlegel); Monacanthidae: *Pampus argenteus* (Euphrasen).	Euryxenous	Layman (1930); Yamaguti (1934); Wang (1982); Li et al. (1989); Shen & Qiu (1995); Kuramochi (2006)

Here, we describe two new species of *Hysterolecitha* from fishes of Moreton Bay, Queensland, Australia. These two new species are essentially cryptic relative to each other in that, despite being clearly distinct genetically while occurring in sympatry, they are effectively morphologically indistinguishable. Although these two species of *Hysterolecitha* infect an overlapping range of host species, they can be partially distinguished on the basis of their host range.

## Materials and methods

### Specimen collection

Fishes were collected from Moreton Bay in southeast Queensland, Australia between 2015 and 2021 via line-fishing and tunnel netting. Fishes were euthanised via an overdose of anaesthetic (AQUI-S®, AQUI-S New Zealand Ltd, Lower Hutt, New Zealand). The gastrointestinal tract was removed and examined for digeneans using the ‘gut wash’ method (Cribb & Bray, 2010). Digeneans were washed in saline, fixed in near-boiling saline, and preserved in 80% ethanol. Multiple specimens were prepared as hologenophores for parallel morphological and molecular analyses (Pleijel et al., 2008).

### Morphological analyses

Specimens were rinsed with distilled water, overstained in Mayer’s haematoxylin, destained in a 1% hydrochloric acid solution, and neutralised in an 1% ammonium hydroxide solution. Specimens were then dehydrated in a graded series of ethanol solutions, cleared in methyl salicylate, and mounted in Canada balsam. Morphometric data were taken using a camera (Olympus SC50) mounted on a compound microscope (Olympus BX-53), and cellSens Standard imaging software. Measurements are in micrometres and are presented in Table 2. Drawings were made using a drawing tube attachment and digitised in Adobe Illustrator. Details of the two new species have been submitted to ZooBank and registered with Life Science Identifiers (LSID) to comply with the recommendations set out in the International Code of Zoological Nomenclature (ICZN, 2012). Specimens are lodged at the Queensland Museum (QM), Brisbane, Queensland, Australia.

**Table 2.** Measurements for *Hysterolecitha melae* **n. sp.** and *H. phisoni* **n. sp.** Values are expressed as a range with the mean in parentheses, in micrometres or as a percentage. Characters highlighted in bold were included in the principal component analysis.

	***Hysterolecitha melae* n. sp.**	***Hysterolecitha phisoni* n. sp.**
**Hologenophores**	19	5
**Paragenophores**	8	9
Body length (BL)	1,232–1,738 (1,482)	1,266–1,925 (1,574)
**Body width**^**1**^	232–451 (334)	252–480 (396)
**Forebody length (FBL)**^**1**^	187–390 (306)	238–545 (404)
FBL % BL	17.1–24.5 (20.7)	24.3–30.9 (26.8)
Hindbody length	732–1,069 (906)	722–1,092 (905)
Left caecum length	920–1,325 (1,157)	947–1,605 (1,268)
Right caecum length	1,014–1,360 (1,211)	953–1,575 (1,260)
Post caecal length	35–107 (67)	47–86 (59)
**Pre-oral lobe length**^**1**^	6–39 (20)	15–28 (20)
**Oral sucker length (OSL)**^**1**^	86–198 (130)	105–194 (141)
**Oral sucker width (OSW)**^**1**^	101–195 (141)	111–200 (152)
**Pharynx length (PL)**^**1**^	42–79 (57)	58–102 (71)
**Pharynx width (PW)**^**1**^	45–86 (65)	49–90 (72)
**Oesophagus length**^**1**^	21–39 (29)	15–61 (35)
**Ventral sucker length (VSL)**^**1**^	181–408 (280)	197–354 (280)
**Ventral sucker width (VSW)**^**1**^	181–411 (293)	179–365 (283)
**VSL:OSL**	1.7–2.6 (2.2)	1.7–2.6 (2)
**VSW:OSW**^**2**^	1.8–2.3 (2.1)	1.8–2 (1.9)
**PL:OSL**^**2**^	0.4–0.5 (0.4)	0.4–0.6 (0.5)
**PW:OSW**^**1,2**^	0.3–0.7 (0.5)	0.4–0.5 (0.5)
**Pre-genital pore length**^**1**^	127–291 (217)	228–373 (285)
**Sinus-sac length**^**1**^	69–129 (106)	73–169 (112)
**Sinus-sac width**^**1**^	48–92 (73)	84–138 (109)
Pars prostatica length	41–56 (49)	53–94 (78)
Pars prostatica width	31–32 (32)	25–44 (34)
**Seminal vesicle length**^**1**^	88–175 (118)	113–206 (161)
**Seminal vesicle width**^**2**^	21–70 (31)	24–70 (53)
**Anterior testis length**^**1**^	58–154 (102)	91–154 (127)
**Anterior testis width**	60–137 (111)	71–183 (142)
**Posterior testis length**^**1**^	66–157 (109)	106–181 (131)
**Posterior testis width**^**1**^	69–166 (122)	119–190 (155)
Distance between testes	8–118 (43)	7–25 (16)
**Posterior testis to ovary length**	82–237 (148)	28–272 (137)
**Pre-ovarian length (PreOL)**^**1**^	639–1,322 (1,061)	749–1,448 (1,100)
**FBL % PreOL**^**2**^	19.2–35.9 (29.6)	30.5–42 (36.8)
**Ovary length (OL)**^**1**^	84–149 (113)	80–139 (113)
OL % BL	6.8–8.7 (7.6)	6.3–8.6 (7.3)
**OL % PreOL**	8–13.9 (10.8)	8.6–13.9 (10.7)
**Ovary width**^**1**^	117–241 (169)	118–193 (158)
Post-ovarian length	273–420 (339)	285–386 (336)
**Egg length**^**1**^	18–23 (21)	22–27 (24)
**Egg width**^**2**^	7–11 (9)	8–11 (9)
Vitellarium field length	160–254 (207)	147–266 (196)
Vitellarium field width	157–321 (221)	156–231 (206)
Post-vitellarium length	114–195 (145)	123–196 (156)

^1^Characters that had significant loadings on principal component 1.

^2^Characters that had significant loadings on principal component 2.

For morphometric analyses, only characters from hologenophores with associated molecular data and paragenophores which were inferred as distinct based on host identity were used. The pre-ovarian length was used as a proxy for body length. To test the significance of some morphometric differences, Welch’s *t*-test was used. A principal component analysis (PCA) was used to further explore the morphometric dataset in R (R Core Team, 2022) using the packages ‘FactoMineR’ (Lê et al., 2008) for the analysis and ‘factoextra’ (Kassambara & Mundt, 2020) for visualisation. As some specimens were damaged or incomplete, not all characters could be measured or included in the analysis. Standard PCA methods are not suitable for incomplete datasets; in this study, deleting individuals or variables with incomplete observations would decrease an already limited dataset and would reduce the statistical power of the analysis. To address this, the package ‘mice’ (van Buuren & Groothuis-Oudshoorn, 2011) was used to impute the missing measurements; these missing values were predicted using multiple imputations based on fully conditional specification, where each variable (or character) is imputed by a separate model. Only specimens with at least 50% of the original measurements were included in the imputation and analysis (*n* = 28). To test the significance of the PCA, the R package ‘PCAtest’ (Camargo, 2022) was used with 1000 bootstrap replications and 1000 random permutations.

### Molecular analysis

Genomic DNA was extracted using a standard phenol/chloroform extraction method (Sambrook & Russell, 2001) and sequence data were generated for one ribosomal DNA (rDNA) marker, the second internal transcribed spacer region (ITS2), and one mitochondrial DNA (mtDNA) marker, the cytochrome c oxidase subunit 1 (*cox*1). These regions were amplified using the primers 3S (5′-GGT ACC GGT GGA TCA CGT GGC TAG TG-3′, Morgan & Blair, 1995) and ITS2.2 (5′-CCT GGT TAG TTT CTT TTC CTC CGC-3′, Cribb et al., 1998) for ITS2, and Dig_cox1Fa (5′-ATG ATW TTY TTY TTY YTD ATG CC-3′, Wee et al., 2017) and Dig_cox1R (5′-TCN GGR TGH CCR AAR AAY CAA AA-3′, Wee et al., 2017) for *cox*1. Amplification was conducted on a TaKaRa PCR Thermal Cycler (TP-690) and amplified DNA was sent to the Australian Genome Research Facility for purification and dual direction Sanger sequencing using the amplification primers.

The ITS2 rDNA and *cox*1 mtDNA datasets were aligned separately in MEGA X (Kumar et al., 2018) using MUSCLE (Edgar, 2004), with UPGMA clustering for iterations 1 and 2. The final dataset for the ITS2 rDNA alignment contained 559 base pairs. The *cox*1 mtDNA alignment was translated (echinoderm/flatworm mitochondrial code) and examined in Mesquite version 3.70 (Maddison & Maddison, 2021) for internal stop codons and to determine the correct reading frame. The alignment was trimmed after the correct reading frame was determined. All codon positions were then tested for non-stationarity in PAUP* version 4.0a (Swofford, 2003), and substitution saturation using the “Test of substitution saturation by Xia et al.” function (Xia et al., 2003; Xia & Lemey, 2009) implemented in DAMBE version 7.2 (Xia, 2018). Non-stationarity and substitution saturation were not detected, and as such, all codons were used in subsequent analyses. The final dataset for the *cox*1 alignment contained 474 base pairs. Neighbour-joining analyses were conducted for each alignment with the following parameters: “Test of Phylogeny = Bootstrap method”, “No. of Bootstrap Replications = 10,000”, “Model/Method = No. of differences”, “Substitutions to Include = d: Transitions + Transversions”, “Rates among Sites = Uniform rates” and “Gaps/Missing Data Treatment = Pairwise deletion”. Pairwise differences for each alignment were estimated using the following parameters: “Variance Estimation Method = None”, “Model/Method = No. of differences”, “Substitutions to Include = d: Transitions + Transversions”, “Rates among Sites = Uniform rates” and “Gaps/Missing Data Treatment = Pairwise deletion”.

## Results

### Overview

**Fig. 1:**
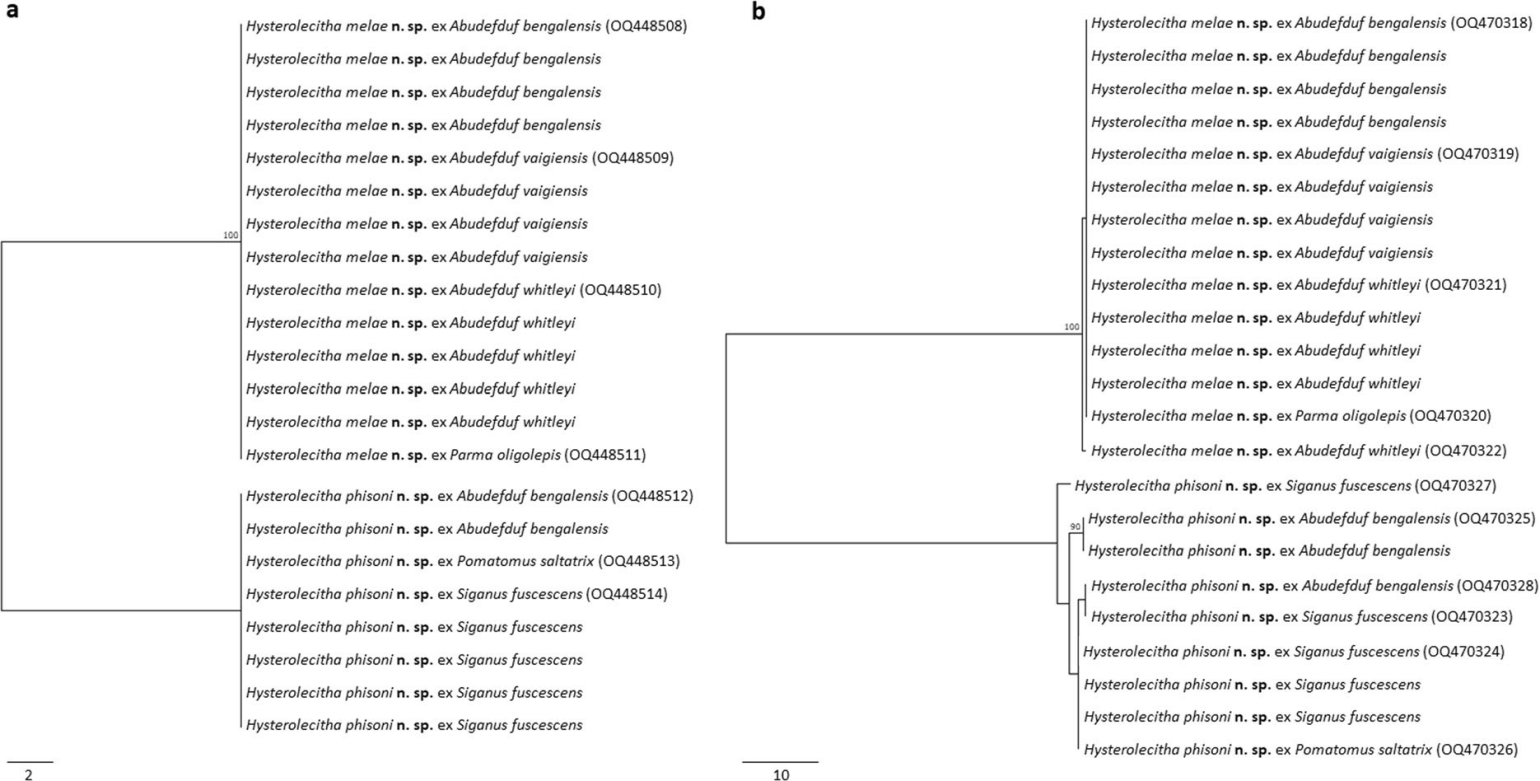
Phylograms based on unrooted neighbour joining analyses of (**a**) ITS2 rDNA and (**b**) *cox*1 mtDNA sequences. Bootstrap support is shown at the node. Scale bar indicates the number of base pair differences.

Specimens morphologically consistent with the genus *Hysterolecitha* were collected from four pomacentrid species, the Bengal sergeant, *Abudefduf bengalensis* (Bloch), the Indo-Pacific sergeant, *A. vaigiensis* (Quoy & Gaimard), Whitley’s sergeant, *A. whitleyi* Allen & Robertson, and the bigscale scaly fin, *Parma oligolepis* Whitley, one pomatomid species, the tailor, *Pomatomus saltatrix* (Linnaeus), and one siganid species, the black rabbitfish, *Siganus fuscescens* (Houttuyn). Initial morphological examination suggested that the collection represented a single species; however, the molecular data demonstrated the clear presence of two forms (Figure 1). A total of 38 ITS2 rDNA and 38 *cox*1 mtDNA sequences were generated. From the ITS2 sequence data, two forms were recognised, differing at 21 base positions. Corresponding *cox*1 sequence data differed at 89–91 base positions. Both forms showed intraspecific variation in the *cox*1 region, with one varying at a single base position, and the second at 1–7 base positions. Re-examination of hologenophores revealed some marginally diagnostic characters that could be used (albeit unreliably) to distinguish between the two forms, specifically the forebody length relative to the body length or the pre-ovarian length, and the development of the terminal genitalia. The two forms are partly biologically distinguished by their host; that is, siganid and pomatomid hosts were only infected by one form, whereas one pomacentrid, *A. bengalensis*, was infected by both.

**Fig. 2:**
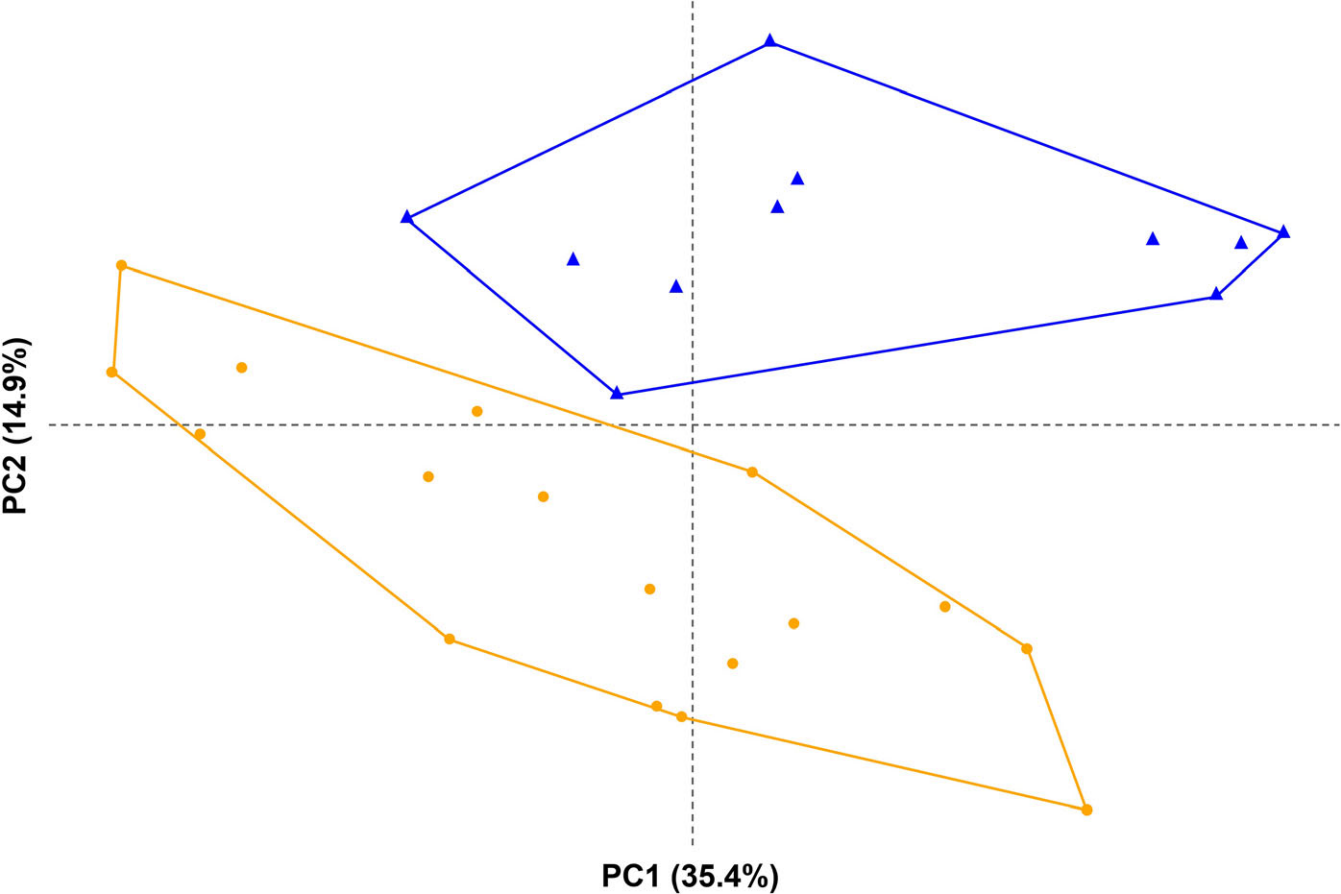
Principal component analysis projection based on the first and second principal components (PC) for the measurements of *Hysterolecitha melae* **n. sp.** hologenophores (orange circles), and *H. phisoni* **n. sp.** hologenophores and paragenophores (blue triangles).

The PCA of the morphometric data revealed a combination of characters which separated the two forms (Figure 2 and Table 2). The PCA permutation tests revealed significant ψ (129.027, *p* = 0) and φ (0.378, *p* <.001) values, indicating that the PCA was biologically significant. Based on cumulative variance and eigenvalues, the first five to eight principal components (PCs) would be retained for further analyses as they accounted for at least 80% of the total variation and had eigenvalues above one (Table 3). However, only PC1 and PC2 were statistically significant and were retained for subsequent analyses. Based on the loading values, a combination of 22 variables contributed significantly to PC1, explaining 35.4% of the total variation (95% confidence interval 30.2–44.8; *p* <.001) and a combination of six variables contributed significantly to PC2, explaining 14.9% of the total variation (95% confidence interval 12.3–21.9; *p* <.001). For list of significant variables see Table 2.

**Table 3.** Eigenvalues and cumulative variance for the first 15 principal components (PCs) based on the principal component analysis performed on a morphometric dataset of *Hysterolecitha melae* **n. sp.** and *H. phisoni* **n. sp.**

**Principal component**	**Eigenvalue**	**Variance (%)**	**Cumulative variance (%)**
PC1	10.97	35.39	35.39
PC2	4.63	14.94	50.33
PC3	2.39	7.71	58.04
PC4	2.09	6.74	64.79
PC5	1.91	6.15	70.94
PC6	1.53	4.93	75.87
PC7	1.44	4.66	80.52
PC8	1.09	3.51	84.04
PC9	0.95	3.05	87.09
PC10	0.82	2.63	89.72
PC11	0.68	2.19	91.91
PC12	0.62	2.01	93.92
PC13	0.38	1.23	95.14
PC14	0.34	1.08	96.22
PC15	0.28	0.89	97.12

The integrated analysis of the morphological and molecular data (as well as the host-specificity) suggest that the two forms of *Hysterolecitha* in the collection represent two species. The forms do not agree with known species of *Hysterolecitha* and are described as new herein.

**Family Lecithasteridae Odhner, 1905**

**Genus *Hysterolecitha*** **Linton, 1910**

Type-species *Hysterolecitha rosea* Linton, 1910 (type by original designation)

**Fig. 3:**
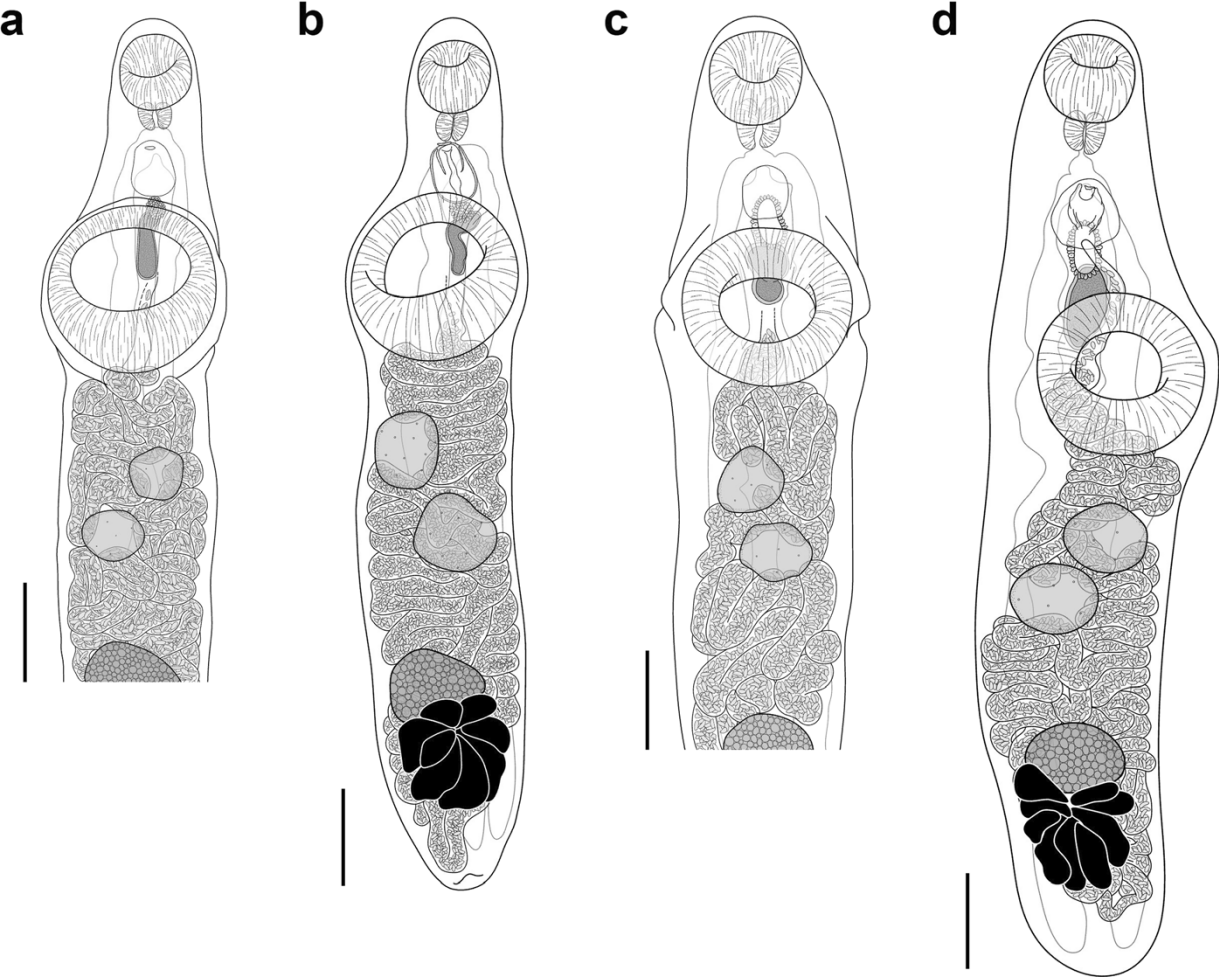
Adult specimens of *Hysterolecitha melae* **n. sp.** and *H. phisoni* **n. sp.** collected from Moreton Bay, Australia. **a**, **b**, *Hysterolecitha melae* **n. sp.** from *Abudefduf bengalensis* (Bloch), (**a**) hologenophore, (**b**) paragenophore, ventral view. **c**, **d**, *H. phisoni* **n. sp.** from *Siganus fuscescens* (Houttuyn), (**c**) hologenophore, (**d**) paragenophore, ventral view. Scale-bars: 200 µm.

**Fig. 4:**
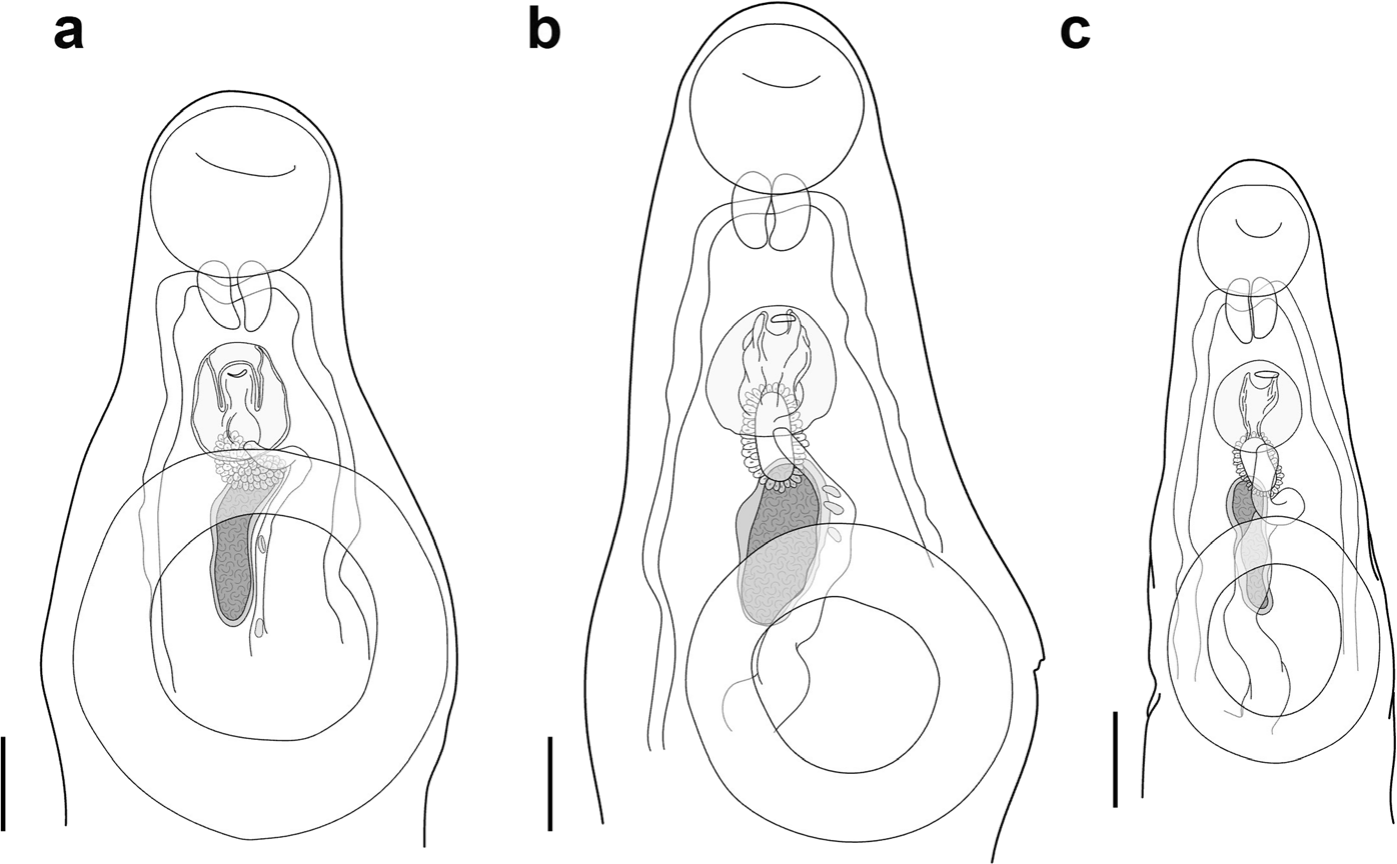
Terminal genitalia of *Hysterolecitha melae* **n. sp.** and *H. phisoni* **n. sp.** collected from Moreton Bay, Australia. **a**, *H. melae* **n. sp.** from *Abudefduf bengalensis* (Bloch), ventral view. **b**, *H. phisoni* **n. sp.** from *Siganus fuscescens* (Houttuyn), ventral view. **c**, *H. phisoni* **n. sp.** from *A. bengalensis*, ventral view. Scale-bars: 100 µm.

***Hysterolecitha melae* n. sp.** (Figures 3a–b and 4a)

*Type-host*: *Abudefduf bengalensis* (Bloch), Bengal sergeant (Pomacentridae).

*Type-locality*: Off Amity Point (27°24′ S 153°25′ E), Moreton Bay, Queensland, Australia.

*Site in host*: Stomach.

*Other hosts*: *Abudefduf vaigiensis* (Quoy & Gaimard), Indo-Pacific sergeant; *Abudefduf whitleyi* Allen & Robertson, Whitley’s sergeant; *Parma oligolepis* Whitley, Bigscale scaly fin (Pomacentridae).

*Prevalence*: 21 of 60 *A. bengalensis*; 7 of 18 *A. vaigiensis*; 8 of 22 *A. whitleyi*; 1 of 2 *P. oligolepis*.

*Type-material*: Holotype (hologenophore, QM G240369), and 16 paratypes (10 hologenophores, QM G240370–74, G240380–83, G240385; six paragenophores, QM G240375-79, G240384).

*Representative DNA sequences*: ITS2 rDNA, 30 identical sequences, four (one of each host/locality combination) submitted to GenBank; *cox*1 mtDNA, 29 sequences, five (one sequence of each genotype/host/locality combination) submitted to GenBank (see Table 4 for accession numbers).

**Table 4.** ITS2 and *cox*1 sequence information (host and GenBank accession numbers) for *Hysterolecitha melae* **n. sp.** and *H. phisoni* **n. sp.**

**Species**	**Host species**	**GenBank Accession number**	
		**ITS2**	***cox*1**
*Hysterolecitha melae* **n. sp.**	*Abudefduf bengalensis* (Bloch)	OQ448508	OQ470318
	*Abudefduf vaigiensis* (Quoy & Gaimard)	OQ448509	OQ470319
	*Abudefduf whitleyi* Allen & Robertson	OQ448510	OQ470321
			OQ470322
	*Parma oligolepis* Whitley	OQ448511	OQ470320
*Hysterolecitha phisoni* **n. sp.**	*Abudefduf bengalensis*	OQ448512	OQ470325
			OQ470328
	*Pomatomus saltatrix* (Linnaeus)	OQ448513	OQ470326
	*Siganus fuscescens* (Houttuyn)	OQ448514	OQ470323
			OQ470324
			OQ470327

*ZooBank registration*:urn:lsid:zoobank.org:act:222B5282-1B30-4E90-82AD-FED139E6C0EB.

*Etymology*: This new species is named for the first author’s sister, Melissa ‘Mel’ Duong, in recognition of her constant support and encouragement.

### Description

Based on 19 hologenophores and eight paragenophores (from all hosts; see Table 2 for measurements). Body elongate, cylindrical, with hindbody wider than forebody, widest at level of mid-ventral sucker. Anterior end of body rounded, tapering distally. Posterior end rounded. Pre-oral lobe usually distinct. Oral sucker globular, subterminal, with anterior half generally broader than posterior half. Ventral sucker rounded, larger than oral sucker, with small papilla-like protrusions on internal surface. Pharynx subglobular, slightly wider than long, overlaps oral sucker dorsally. Oesophagus shorter than pharynx. Intestinal bifurcation in mid-forebody. Caeca irregularly narrow, dorsal to uterus, testes, ovary and vitellarium, reach close to posterior extremity. Genital pore a transverse ellipse, median. Sinus-sac claviform, longer than wide, proximal end typically borders anterior margin of ventral sucker, encloses well-developed sinus-organ. Pars prostatica oval, represented by cluster of prostatic cells. Seminal vesicle saccular, elongate, extends to mid-level of ventral sucker. Testes irregularly oval, oblique, generally separated, in anterior half of hindbody. Ovary transversely oval, in posterior half of hindbody. Seminal receptacle rounded, post-ovarian, typically obscured by vitellarium lobes, uterus or eggs. Juel’s organ not detected. Uterus fills most of hindbody; coils typically do not extend anteriorly past posterior margin of ventral sucker. Metraterm not differentiated from uterus. Eggs numerous, small, tanned, operculate, without bipolar filaments, tightly packed together in uterine coils. Vitellarium comprised of seven to eight compact digitiform lobes, radiating from central point immediately posterior to ovary. Excretory arms tubular, typically obscured by uterus and eggs, unite dorsally at level of pharynx. Excretory pore terminal.

***Hysterolecitha phisoni* n. sp.** (Figures 3c–d and 4b–c)

*Type-host*: *Siganus fuscescens* (Houttuyn), Black rabbitfish (Siganidae).

*Type-locality*: Off Green Island (27°25′ S 153°14′ E), Moreton Bay, Queensland, Australia.

*Site in host*: Stomach.

*Other hosts*: *Abudefduf bengalensis* (Bloch), Bengal sergeant (Pomacentridae); *Pomatomus saltatrix* (Linnaeus), Tailor (Pomatomidae).

*Other localities*: Off Garden Island (27°37′ S 153°20′ E) and off Amity Point (27°24′ S 153°25′ E), Moreton Bay, Queensland, Australia.

*Prevalence*: 3 of 60 *A. bengalensis*; 1 of 18 *P. saltatrix*; 6 of 31 *S. fuscescens*.

*Type-material*: Holotype (QM G240386), and 10 paratypes (five hologenophores, QM G240387–88, G240393–94, G240396; five paragenophores, QM G240389–92, G240395).

*Representative DNA sequences*: ITS2 rDNA, eight identical sequences, three (one of each host/locality combination) submitted to GenBank; *cox*1 mtDNA, nine sequences, six (one sequence of each genotype/host/locality combination) submitted to GenBank (see Table 4 for accession numbers).

*ZooBank registration*: urn:lsid:zoobank.org:act:4BC2369B-5BAF-4016-A16C-BB20E1526A09.

*Etymology*: The new species is named for the first author’s partner, Brody Phi Son Ly, in recognition of his constant support and encouragement.

### Description

Based on five hologenophores (from *A. bengalensis* and *S. fuscescens*) and nine paragenophores (from *S. fuscescens*; see Table 2 for measurements). Body elongate, cylindrical, widest at level of mid-ventral sucker. Anterior end of body rounded, slightly tapering distally. Posterior end rounded, blunt. Pre-oral lobe usually distinct. Oral sucker globular, subterminal, with anterior half generally broader than posterior half. Ventral sucker in proximal region of anterior half of body, rounded, larger than oral sucker, with small papilla-like protrusions on internal surface. Pharynx subglobular, slightly longer than wide, overlaps oral sucker dorsally. Oesophagus shorter than pharynx. Intestinal bifurcation in mid-forebody. Caeca irregularly narrow or wide, dorsal to uterus, testes, ovary and vitellarium, reach close to posterior extremity. Genital pore a transverse ellipse, median. Sinus-sac rounded, generally slightly longer than wide, encloses poorly developed sinus-organ. Pars prostatica oval or reniform, lined by prostatic cells. Seminal vesicle saccular, dorsally overlaps anterior margin of ventral sucker. Testes oval, oblique, generally contiguous, in anterior half of hindbody. Ovary transversely oval, in posterior half of hindbody. Juel’s organ and seminal receptacle not detected. Uterus fills most of hindbody; coils can extend dorsally past posterior margin of ventral sucker. Metraterm not differentiated from uterus. Eggs numerous, small, tanned, operculate, without bipolar filaments. Vitellarium comprised of seven to eleven compact digitiform lobes, radiating from central point immediately posterior to ovary. Excretory arms tubular, typically obscured by uterus and eggs, unite dorsally at level of pharynx. Excretory pore terminal.

### Remarks

**Fig. 5:**
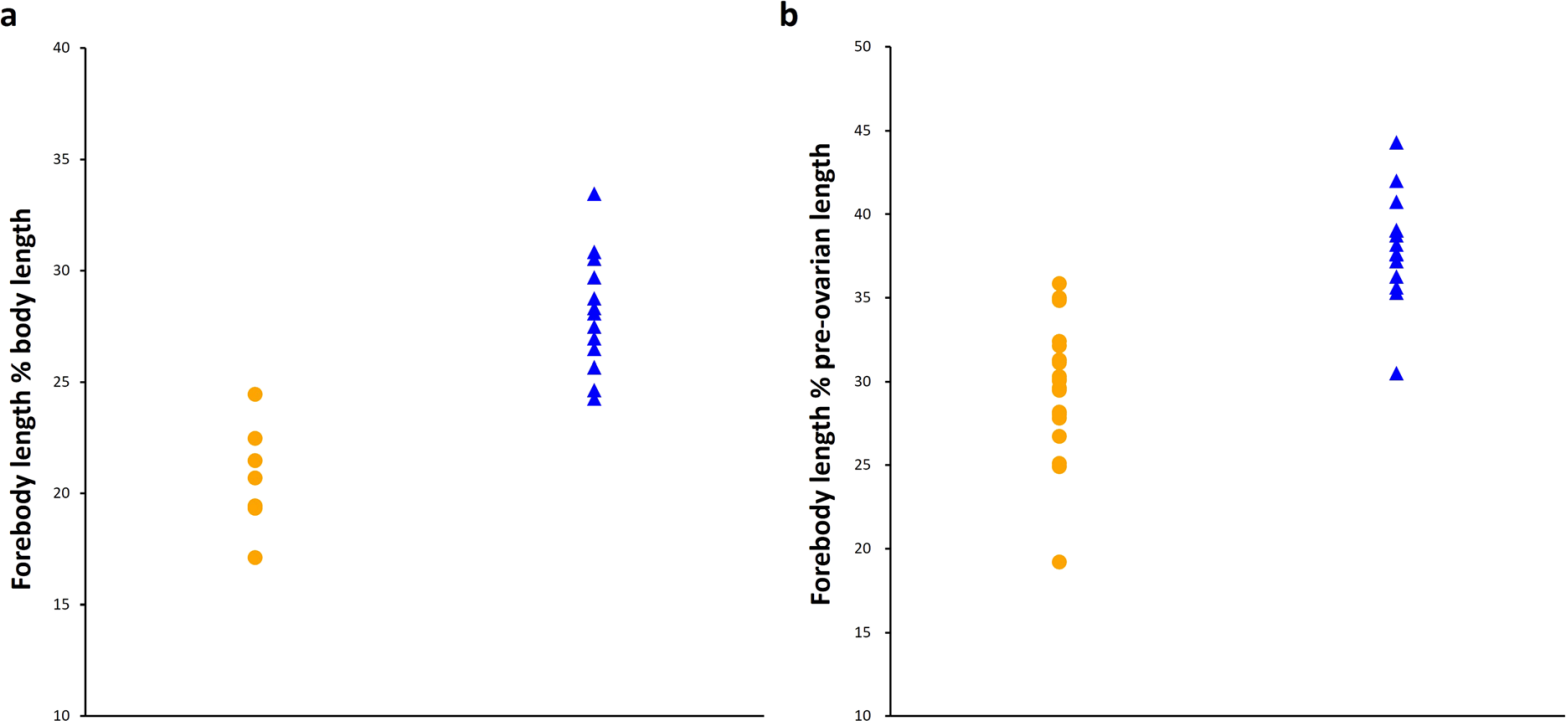
Plot of the forebody lengths relative to (**a**) body lengths and (**b**) pre-ovarian lengths of *Hysterolecitha melae* **n. sp.** (orange circles, **a**, paragenophores only, **b**, hologenophores and paragenophores) and *H. phisoni* **n. sp.** (blue triangles, **a** and **b**, hologenophores and paragenophores).

The current collection of *Hysterolecitha* specimens sampled from Moreton Bay fishes represents a case of sympatric, cryptic species that are associated with a partial overlap in host species ranges. Representative specimens from each individual host were prepared as a hologenophore and the subsequent genetic data associated with each hologenophore was used to tentatively identify paragenophores. This process resulted in collections of worms from single host individuals being identified as the same species. As the two species are essentially cryptic, we acknowledge that this division may not be completely reliable; definitive species identification is presently dependent on genetic data (and, partly, host identity). Based on this genetic delineation, specimens were compared morphologically to find a basis of distinction. There is a significant difference in the forebody length relative to: a) body length and b) pre-ovarian length between specimens of *H. melae* **n. sp.** (a: Mean, M = 20.7, Standard Deviation, SD = 2.4; b: M = 29.6, SD = 4) and *H. phisoni* **n. sp.** (a: M = 28.1, SD = 2.6; b: M = 38, SD = 3.3); a: *t*(13) = -6.369, *p* <.001, b: *t*(31) = -6.676, *p* <.001. Specimens of *H. melae* **n. sp.** generally have shorter forebodies relative to their body length and pre-ovarian length, whereas *H. phisoni* **n. sp.** specimens generally have longer forebodies; however, this pattern is not completely consistent and is not observed across all specimens (Figure 5). The specimens also differed in the development of their terminal genitalia; *H. melae* **n. sp.** generally has a well-developed sinus-organ and a smaller pars prostatica than *H. phisoni* **n. sp.** which has a poorly developed sinus-sac and a larger pars prostatica.

## Discussion

### Species delineation

Bray et al. (2022) proposed a set of criteria for trematode species delineation which requires reciprocal monophyly in the most discriminatory molecular markers in combination with either morphological differences relative to other taxa or distinctions in host species ranges relative to those of closely related taxa. The recognition of two species of *Hysterolecitha* here is based on ITS2 and *cox*1 sequence data, and morphometric analyses that showed that the specimens formed distinct clusters. The strong reciprocal monophyly is the key evidence in justifying the recognition of two species. The two species are also partly distinguished by host range; specimens of *H. melae* **n. sp.** were found in only four pomacentrid species whereas *H. phisoni* **n. sp.** was found commonly in a siganid, uncommonly in a pomacentrid, and rarely in a pomatomid. Therefore, the interpretation of these data is that the present collection of *Hysterolecitha* from Moreton Bay comprises two genetically distinct species that are essentially morphologically cryptic.

Relative to the 20 marine species of *Hysterolecitha*, *H. melae* **n. sp.** and *H. phisoni* **n. sp.** are morphologically most similar to *H. heronensis* (reported from pomacentrids), *H. lintoni* Srivastava, 1939 (reported from an ariid), *H. nahaensis* (reported from several families including pomacentrids and siganids), *H. rosea* Linton, 1910 (reported mostly from acanthurids), and *H. teuthis* Nagaty, 1956 (reported from a siganid). However, both the new species differ from *H. heronensis* by a having smaller sucker width ratio (1:1.8–2.3 and 1:1.8–2.0 *vs* 1:2.65), a short sinus-sac (*vs* elongated) and compact irregular digitiform vitelline lobes (*vs* elongated digitiform lobes) and from *H. lintoni* by having an oesophagus present (*vs* absent) and a saccular seminal vesicle (*vs* constricted). The two new species differ from *H. nahaensis* by having irregular digitiform vitelline lobes (*vs* rounded lobes), and from *H. rosea* and *H. teuthis* by having a saccular seminal vesicle (*vs* sinuous). Of the remaining species of *Hysterolecitha*, the two new species differ from *H. acanthuri* Annereaux, 1947, *H. palani* Yamaguti, 1970, *H. sogandaresi* Nahhas & Cable, 1964, and *H. trilocalis* King & Noble, 1961 in having a saccular seminal vesicle (*vs* sinuous), from *H. arii* Wang, 1982, *H. blepsiae* Layman, 1930, and *H. vitellograndis* (Layman, 1930) Skrjabin & Guschanskaja, 1954 in having digitiform vitelline lobes (*vs* rounded or club-shaped), and from *H. brasiliensis* de Oliveira, Amato & Knoff, 1988, *H. crassivesiculata* Bravo-Hollis, 1956, *H. flaticaudata* Bilqees, Feroze & Shaukat, 2004, and *H. indonesiana* Machida, 1996 in having smaller eggs (19–27 × 7–11 *vs* 24–43 × 16–22, 34–40 × 18–22, 34–42 × 21–31 and 32–39 × 18–22, respectively). The two new species differ from *H. chirocentri* Ku & Shen, 1964 in possessing a united vitellarium (*vs* divided into two clusters), from *H. elongata* Manter, 1931 in having a shorter post-ovarian region (*vs* elongated), and from *H. progonimus* Ku & Shen, 1964 in having a seminal vesicle that dorsally overlaps the anterior end of the ventral sucker (*vs* a seminal vesicle that terminates at the level of the mid-forebody). Finally, the two new species differ from *H. soniae* León-Règagnon, Perez-Ponce de Leon & Lamothe-Argumedo, 1997 in possessing an oval pars prostatica (*vs* sinuous).

The genetic differences reported here for *H. melae* **n. sp.** and *H. phisoni* **n. sp.** are generally comparable to other combinations of cryptic species. Recent studies have reported genetic differences of up to 21 base positions in the ITS2 region and up to 53 base positions in the *cox*1 region for cryptic species of lepocreadiids from the GBR (Bray et al., 2018; Bray et al., 2022) and monorchiids from the GBR and Japan (Wee et al., 2022). However, the genetic differences among some closely related, non-cryptic species has been reported to be much lower. For example, studies on morphologically distinguishable species of bivesiculids (Trieu et al., 2015; Cribb et al., 2022) and lepocreadiids (Bray et al., 2018; Bray et al., 2022) from GBR fishes have reported differences at one to two base positions in the ITS2 region and up to 52 base positions in the *cox*1 region. We conclude that use of a ‘yardstick’ approach to the interpretation of the significance of levels of molecular distinction is problematic. Instead, interpretations are best made on a case by case basis in the light of all available evidence. Here, we interpret the evidence as clearly indicating the presence of two species.

### The genus *Hysterolecitha*

Previous reports of species of *Hysterolecitha* have been overwhelmingly based on morphometric data in isolation. A search for *Hysterolecitha* sequence data on GenBank returned only a single result, an 18S sequence of *H. nahaensis* (from an unknown host and locality), generated as part of a large phylogenetic study of the Hemiuroidea (Blair et al., 1998). This lack of genetic data associated with reports (and descriptions) makes reliable species delineation and identification difficult, especially for species as morphologically cryptic as *H. melae* **n. sp.** and *H. phisoni* **n. sp.** Without supporting genetic data, the current collection of Moreton Bay *Hysterolecitha* specimens would certainly have been considered a single euryxenous species with marginal intraspecific morphological variation. The genetic data, however, clearly indicates that the new specimens comprise two species with different forms of host-specificity (euryxenous and stenoxenous).

Of the known marine *Hysterolecitha* species, five have been reported from more than one locality, with *H. nahaensis* [see Yamaguti (1942), Yamaguti (1953), Parukhin (1989) and Zhokhov et al. (2018)] and *H. rosea* [see Linton (1910) and Wang (1982)] being reported as the most widespread. However, like the majority of *Hysterolecitha* species, the two new species are known from only a single locality, but unlike for most species of the genus, there is some evidence of absence. *Hysterolecitha melae* **n. sp.** and *H. phisoni* **n. sp.** have not been detected at other Australian localities, specifically the GBR, where two other known species (*H. heronensis* and *H. nahaensis*) have been found (Bray et al., 1993; Barker et al., 1994; Sun et al., 2012). While the distribution of *Pomatomus saltatrix* does not indicate it would be found in the GBR (Bray, 2022), the pomacentrid and siganid hosts of *H. melae* **n. sp.** and *H. phisoni* **n. sp.** have broad distributions that encompass the GBR (Randall et al., 1998; Parmentier & Frédérich, 2016; Bray, 2020). However, based on extensive collecting in Queensland waters for over 20 years, we have not detected either of the two new species (or the known species) outside of their respective known localities. That is, *H. melae* **n. sp.** and *H. phisoni* **n. sp.** have not been found in the GBR, and *H. heronensis* and *H. nahaensis* have not been found from Moreton Bay, despite the presence of suitable hosts for each species at these locations.

### Cryptic species complexes in the Hemiuroidea

Digeneans have been reported to have some of the highest levels of cryptic diversity relative to other helminth taxa (Pérez-Ponce de León & Poulin, 2018). The findings of cryptic diversity here extend a growing list of cases within the Hemiuroidea. One reported case of cryptic hemiuroids was by Carreras-Aubets et al. (2011) who described a new species related to the lecithasterid *Aponurus laguncula* Looss, 1907. The new species was recognised for combined molecular, morphological and host distinctions. The new species was not strictly morphologically cryptic relative to *A. laguncula*, but it was sufficiently inconspicuous to have escaped recognition for over 100 years since the description of *A. laguncula*, although that species was suspected to constitute a species complex due to its euryxenous host-specificity and wide geographical distribution (Bray & MacKenzie, 1990; Bray et al., 1993). Another case was for the genus *Hirudinella* de Blainville, 1828 (Hirudinellidae). For this genus, the delineation of species is made challenging by the huge size of the specimens and the lack of reliable characters to separate species (Gibson & Bray, 1979); as a result, many nominal *Hirudinella* species are no longer recognised. Calhoun et al. (2013) used ITS1, ITS2 and 28S rDNA sequence data to show that specimens consistent with *Hirudinella ventricosa* (Pallas, 1774) Baird, 1853 comprised four species. Despite the clarity of the molecular data, only two of the species (*H. ventricosa* and *H. ahi* Yamaguti, 1970) could be formally recognised; two other species were not named due to limitations associated with the morphological features and additional genetic data. Recent molecular work on morphologically similar specimens of *Lecithaster* Lühe, 1901 (Lecithasteridae) collected from fishes belonging to multiple families has resulted in the recognition of two species that had been synonymised principally on morphometric similarity (Atopkin et al., 2020). *Lecithaster sayori* Yamaguti, 1934 and *L. salmonis* Yamaguti, 1934 [previously synonymised with *L. stellatus* Looss, 1907 (see Manter & Pritchard, 1960) and *L. gibbosus* (Rudolphi, 1802) Lühe, 1901 (see Margolis & Boyce, 1969), respectively], were shown to be genetically distinct based on ribosomal markers. Most recently, adult and cercarial specimens morphologically consistent with *Derogenes varicus* (Müller, 1784) Looss, 1901 (Derogenidae) were shown to be genetically distinct, forming up to four lineages based on ribosomal and mitochondrial markers that were associated with different hosts and localities (Olson et al., 2003; Sokolov et al., 2021; Krupenko et al., 2022). Although the genetic distinctions are clear, the lack of corresponding adult morphological specimens has hindered the naming of these lineages as new species. The current findings of cryptic species of *Hysterolecitha* here are broadly consistent with the previous studies reviewed above. As is frequently the case, the distinct species here were largely associated with different host taxa. The most difficult problem of cryptic species, where combinations of species occur in the same host and the same locality (as partly occurred here), is not commonly reported.

## Data Availability

The data that support the findings of this study are available from the corresponding author upon reasonable request.
